# U.S. medical students who engage in self-care report less stress and higher quality of life

**DOI:** 10.1186/s12909-018-1296-x

**Published:** 2018-08-06

**Authors:** Erin E. Ayala, Jeffrey S. Winseman, Ryan D. Johnsen, Hyacinth R. C. Mason

**Affiliations:** 1 Department of Counseling Psychology, Saint Mary’s University of Minnesota, 2500 Park Avenue, Minneapolis, MN 55404 USA; 2 Department of Psychiatry, Albany Medical College, 25 Hackett Blvd, Albany, NY 12208 USA; 3 Department of Emergency Medicine, HealthPartners Institute Regions Hospital, 640 Jackson Avenue, Saint Paul, MN 55101 USA; 4 Departments of Medical Education and Family and Community Medicine, Albany Medical College, 47 New Scotland Avenue, 12208 Albany, NY, USA

**Keywords:** Medical students, Medical education, Self-care, Stress, Quality of life, Health promotion

## Abstract

**Background:**

Research on student wellness has highlighted the importance of self-care for medical students; however, scholars have yet to identify the extent to which self-reported engagement in self-care behaviors is associated with attenuation of the negative relationship between stress and quality of life during the initial years of medical education.

**Methods:**

Using a self-report survey designed to measure self-care, perceived stress, and quality of life, we hypothesized that self-care would moderate the relationship between stress and psychological quality of life in medical students, as well as stress and physical quality of life.

An online questionnaire was completed by 871 medical students representing 49 allopathic medical colleges throughout the U.S. between December 2015 and March 2016. The survey assessed perceived stress, self-care, quality of life and a variety of demographic variables. Regression analyses were used to assess interaction effects of self-care on the relationships between stress and quality of life.

**Results:**

Self-reported engagement in self-care appeared to moderate the relationships between perceived stress and both physical (*p* < .001) and psychological (*p* = .002) quality of life. As the level of reported engagement in self-care increased, the strength of the inverse relationship between perceived stress and both physical and psychological quality of life appeared to weaken.

**Conclusions:**

Our findings suggest that self-reported engagement in self-care activities is associated with a decrease in the strength of the relationship between perceived stress and quality of life in medical students. Students who disclose utilizing a multitude of self-care practices throughout their training may also sustain greater resiliency and lower risk for higher levels of distress during medical education.

## Background

Medical training is a stressful time for students worldwide and has been shown to be associated with high rates of burnout, anxiety, and depression, adversely affecting physicians throughout their careers, particularly in the United States [1–7]. One approach to reducing stress and promoting well-being during medical education is to engage in effective self-care [8, 9]. Self-care is a multifaceted health behavior unique to each person, and includes nutrition, physical activity, interpersonal relations, spiritual growth, health responsibility, and stress management [10]. Recent mixed method studies of student opinions both at one medical school and nationally suggest that students tend to use a wide array of highly individualized stress reduction behaviors, and that students may view personal health and relationships as the most important factors affecting well-being during medical school [11, 12]. However, studies utilizing self-report measures also consistently show that U.S. medical students often pay inadequate attention to self-care [13–15].

Complex demands placed on students during medical education often make it difficult to prioritize the time necessary for maintaining personal well-being [16]. Research examining medical students’ attitudes toward self-care during medical school suggests that aspects of the medical education culture as a whole (e.g., increased pressure, time constraints, help-seeking stigma) often prevent educators and students from being able to advocate and care for themselves adequately [17–20]. Many schools have worked to inculcate self-care in students by implementing programs that help students manage daily stressors [20–26]; however, while research shows that many of these programs improve quality of life in medical students, self-care remains difficult to operationalize within an educational competency.

Although studies of medical student well-being show that while self-care and health promotion initiatives are associated with improved quality of life [9, 20], students’ level of perceived stress is correlated with poorer resilience [27], as well as increased mental and physical health needs [14, 28]. Quality of life has been defined as the physical, psychological, social, and/or environmental well-being of an individual with respect to his/her goals, standards, and culture [29]. Traditionally, investigations into the benefits of positive self-care during medical education have focused on how one or more separate aspects of self-care such as mindfulness, exercise, nutrition, or sleep affects students’ stress and/or quality of life [23, 24, 26]. Yet, studying self-care based on one of the above single aspects of behavior as opposed to an overarching pattern of self-directed behavior that includes multiple facets of well-being (e.g., stress management, health responsibility, physical activity, nutrition, spiritual growth, interpersonal relations [10]) may limit our ability to synthesize these research findings. Examining the specific relationships between medical student stress, self-care and quality of life using a comprehensive conceptualization of self-care may help to more accurately identify the associations between these common variables and offer a more consistent way for researchers to compare various self-care interventions.

### Research questions, aims and hypotheses

Despite implementation of programs designed to promote student wellness [20–26], researchers have yet to empirically examine the extent to which higher reported levels of self-care may decrease the strength of the relationship between stress and quality of life in medical students. To address this gap in the literature, we tested U.S. students’ self-reported engagement in self-care on the relationships between perceived stress and quality of life. Using measures designed to capture the multifaceted nature of both self-care and quality of life, we hypothesized that higher reported involvement in self-care would decrease the strength of the relationship between medical student stress and both physical and psychological quality of life. More specifically, we hypothesized that the interaction between stress and reported engagement in self-care would reveal a moderating, or protective, effect of engagement in self-care in light of the negative effects of stress on quality of life during medical education.

## Methods

### Participants and procedures

Participant data were used from a dataset containing 871 medical student responses to a national, multi-component wellness survey [11]. A respondent driven sampling method was utilized to create the database [30]. Administrators from all Liaison Committee on Medical Education (LCME) accredited medical institutions in the U.S. were contacted between December 2015 and March 2016 and asked to invite medical students to participate in an optional and confidential survey assessing stress, health, and well-being. Students were also recruited via professional listservs, student organizations, and institutional administrations. Qualtrics™ (Provo, Utah, United States) online platform was used to record data.

Responses from medical students at all stages of education were included. Medical education in the United States typically includes two pre-clinical years followed by a third year of core clinical service rotations and a final fourth year of elective and non-core specialty rotations. Thus, participants who reported they were in the third year of allopathic medical schools were understood to be in their core clinical year, often identified as the most vulnerable period for U.S. medical students [31]. The research team attained approval by the Institutional Review Board (IRB) in addition to a certificate of confidentiality from the National Institutes of Health (NIH).

### Study measures

#### Stress

The Perceived Stress Scale is a 14-item instrument that measures how often participants have experienced feelings associated with stress in the past month on a scale of 0 (*never*) to 4 (v*ery often*) [32, 33]. Total scores range from 0 to 56; higher scores represent higher levels of perceived stress. The measure has strong internal reliability (Cronbach’s *α*s = .84–.86), test-retest reliability (*r*_*tt*_ = .85), concurrent validity, and predictive validity for depressive and physical symptomatology for college students and the general population. Internal consistency for the current investigation was strong (*α* = 0.91; Table 1).

**Table 1:** Intercorrelations, Reliabilities, and Descriptive Statistics for Study Variables

	Combined Sample		Men		Women		α	Stress	Self-Care	Physical QoL
	*M*	*SD*	*M*	*SD*	*M*	*SD*				
Stress	27.49	7.76	26.35	8.12	28.05	7.56	.91			
Self-Care	2.54	0.43	2.48	0.40	2.57	0.43	.93	−.60*		
Physical QoL	55.68	12.11	55.35	12.16	55.96	11.90	.74	−.59*	.60*	
Psychological QoL	62.23	12.11	62.06	14.71	62.56	13.25	.78	−.61*	.67*	.45*

*N* = 871. *M =* Mean, *SD* = Standard Deviation, α = Cronbach’s alpha. *p < .001. QoL = Quality of Life

#### Self-care

The Health Promoting Lifestyle Profile II (HPLPII) is a 52-item measure that operationalizes self-care as a multifactorial construct and includes six sub-scales: Nutrition, Physical Activity, Interpersonal Relations, Spiritual Growth, Stress Management, and Health Responsibility [34]. The scale was selected because it includes a wide variety of health promoting behaviors, many of which have been endorsed by medical students in mixed methods studies [11, 12]. Using a 4-point Likert-type scale (1 = *never* to 4 = *routinely*), questions assess the extent to which participants engage in health-promoting behaviors in each of the six categories. Means for both total and subscale scores are computed, thus scores may range from 1 to 4. Higher scores represent higher levels of self-care. In addition to a strong conceptual framework, the scale includes strong internal reliability (*α* = .90), test-retest reliability (*r*_tt_ = .81–.91), and convergent validity with other health measures [34, 35]. Internal reliability for the current investigation was strong (*α* = .93; Table 1).

#### Quality of life

The World Health Organization’s (WHO) 26-item quality of life measure includes four scales that examine an individual’s physical, psychological, social, and environmental well-being using a 5-point Likert type scale [29]. The scale has been normed on an international sample of adults from over 23 countries, has strong construct validity, and is used frequently in public and behavioral health. The inventory treats each of the four scales independently. The physical and psychological quality of life subscales were used in this investigation. Scores are converted to a scale ranging from 0 to 100. Higher scores represent higher quality of life. Cronbach’s alphas for quality of life subscales in this study ranged from .74 to .78 (Table 1).

#### Demographic questionnaire

Participants were asked to record their age, gender, sexual orientation, racial background, first generation student status, marital status, and year in medical school.

### Statistical analysis

Before conducting inferential analyses, we examined missing data, discrepant data, and assumptions of normality, linearity, multicollinearity, and homogeneity of variance [36]. In order to account for difficulties of assessing moderation effects in applied settings (e.g., small effect sizes; increased error) [37], we performed an a priori power analysis using a small effect size (*f*^2^ = 0.02), conservative alpha rate (*α* = .01), and power of 0.80. To detect a small moderation effect, at least 779 participants were needed in the study.

Before analyzing findings, we examined internet protocol (IP) addresses and demographic information to identify potential duplicates in the data. Participants who had duplicate IP addresses with matching demographic information were to be removed from the dataset. No duplicate cases were identified.

Two regression analyses were performed using the Hayes PROCESS macro (2013) in IBM SPSS statistical software (SPSS, Inc., Chicago, IL, USA). Perceived stress and self-care served as independent variables, the interaction term served as the moderator, and psychological and physical quality of life served as the dependent variable in each respective analysis. A significant interaction term signified a statistically significant moderating effect of reported engagement in self-care on the relationship between perceived stress and quality of life.

## Results

### Participants

Students (*N* = 871) representing 49 LCME accredited medical institutions in the U.S. completed the survey. Participants ranged in age from 20 to 45 and represented all four years of undergraduate medical training. Demographic information and descriptive statistics are shown in Tables 1 and 2.

**Table 2:** Demographic Characteristics of Sample

	Current Sample		Total Enrollment in U.S.*	
	*n*	%	*n*	*%*
Gender				
Women	540	60.1	40,583	46.9
Men	307	34.2	46,027	53.1
Year in Medical School				
First	264	29.4	20,627	25.6
Second	224	24.9	20,343	25.3
Third	190	21.2	20,055	24.9
Fourth	173	19.3	19,517	24.2
Race/Ethnicity				
Non-Hispanic White	639	71.2	46,841	53.1
Asian	71	7.9	18,430	20.9
African American	37	4.1	5856	6.63
Hispanic	58	6.5	5344	6.1
Native American	4	0.4	96	0.1
More than one race	31	3.5	6740	7.6
Sexual Orientation				
Heterosexual/Straight	794	85.1	13,447	94.3
Gay or Lesbian	21	2.3	484	3.4
Bisexual	42	4.7	327	2.3
Pansexual	10	1.1	N/A	N/A
Marital Status				
Single	544	60.6	10,612	73.9
Living with partner	82	9.1	N/A	N/A
Married	150	16.7	3532	24.6
Engaged	67	7.5	N/A	N/A
Separated/Divorced	9	1.0	129	0.9
First Generation College Graduate				
Yes	123	13.7	9493	15.0
No	727	81.0	53,945	85.0

*N* = 871. *Data from Association of American Medical Colleges [60], AAMC Medical School Graduation Questionnaire [65], and Brewer & Grbic [66]

### Interaction effects between self-care and stress on quality of life

**Fig. 1:**
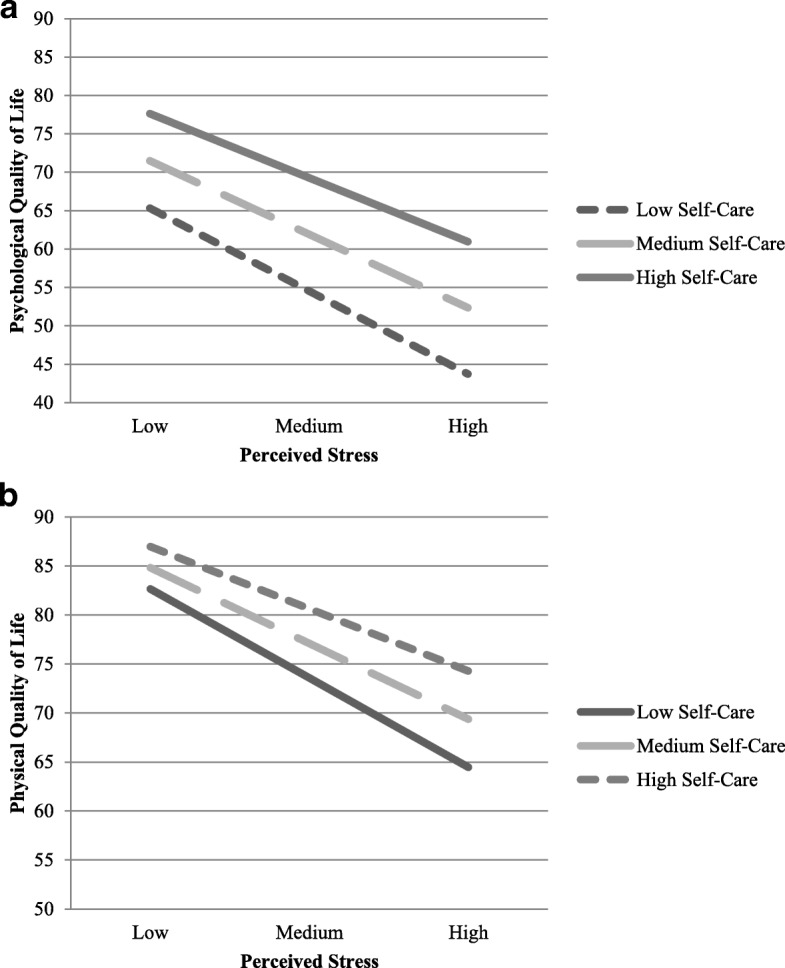
**a-b** Simple slopes of stress predicting psychological and physical quality of life for 1 standard deviation below the mean of self-care, the mean of self-care, and 1 standard deviation above the mean of self-care

Perceived stress, reported engagement in self-care, and the interaction between stress and engagement in self-care accounted for 64.7% of the variance in psychological quality of life, *F*(3, 764) = 465.89, *p* < .001 (Table 3). Perceived stress was significantly associated with decreased psychological quality of life when accounting for other variables in the model, *β* = −.50, 95% CI [−.55, −.44], *p* < .001. Reported engagement in self-care was positively associated with psychological quality of life, *β* = 0.38, 95% CI [.32, .44], *p* < .001. Finally, the significant interaction term suggested that reported engagement in self-care activities significantly decreased the strength of the relationship between perceived stress and psychological quality of life, *β* = 0.06, 95% CI [.02, .10], *p* = .002 (Fig. 1). That is, as reported engagement in self-care increased, the strength of the inverse relationship between perceived stress and psychological quality of life decreased.

**Table 3:** Coefficients and Parameters for Regression Model

		*β*	*SE β*	*t*	*p*	95% CI
Psychological Quality of Life	Constant	.04	.02	1.56	.120	[−.01, .09]
	Self-Care	.38	.03	14.15	< .001	[.33, .44]
	Stress	−.50	.03	−18.20	<.001	[−.55, −.44]
	Interaction	.06	.02	3.09	.002	[.02, .10]
	R^2^ = .647					
Physical Quality of Life	Constant	.06	.03	2.09	.040	[.00, .12]
	Self-Care	.22	.03	6.77	< .001	[.16, .29]
	Stress	−.49	.03	−14.71	<.001	[−.56, −.43]
	Interaction	.09	.03	3.47	<.001	[.04, .14]
	R^2^ = .463

Second, the full model including perceived stress, self-care, and the interaction between stress and self-care explained 46.3% of the variance in physical quality of life, *F*(3, 768) = 220.54, *p* < .001 (Table 3). Perceived stress was significantly and inversely associated with physical quality of life when controlling for self-care and the interaction term, *β* = −.49, 95% CI [−.56, −.43], *p <* .001. Additionally, reported engagement in self-care was significantly associated with physical quality of life, *β* = 0.22, 95% CI [.16, .29], *p* < .001. Finally, the interaction between stress and reported engagement in self-care was also significant, suggesting engagement in self-care decreased the strength of the relationship between perceived stress and physical quality of life, *β* = 0.09, 95% CI [.04, .14], *p* < .001 (see Fig. 1). More specifically, higher reported levels of self-care were associated with a weaker inverse relationship between perceived stress and physical quality of life.

### Post hoc analyses

We performed post hoc analyses on the demographic variables to examine differences in stress, self-care, and quality of life across demographic groups.

#### Self-care

Men had significantly lower levels of self-care than women, *t*(777) = − 2.94, *p* = .003, *d* = .21. Non-Hispanic White students (*M* = 2.56, *SD* = 0.42) had significantly higher levels of reported self-care than students of other racial or ethnic backgrounds (*M* = 2.44, *SD* = .42), *t*(318.07) = 3.49, *p* = .001, *d* = 0.39. Fourth year medical students had significantly higher reported levels of self-care in comparison to first year medical students and third year medical students (*p* < .001), and second-year medical students had significantly higher levels of self-care in comparison to third year medical students (*p* = .007).

#### Stress

Women reported significantly more perceived stress than men, *t*(830) = 3.05, *p* = .002, *d* = .21. Non-Hispanic White students (*M* = 27.12, *SD* = 7.77) had significantly lower levels of perceived stress than students of other racial or ethnic backgrounds (*M* = 28.38, *SD* = 7.85), *t*(352.71) = − 2.00, *p* = .047, *d* = − 0.21. There were also significant differences in perceived stress based upon year of study, *F*(3,833) = 13.564, *p* < .001. That is, fourth year medical students reported significantly lower levels of stress in comparison to first year (*p* < .001), second year (*p* < .001), and third year students (*p* < .001).

#### Quality of life

Non-Hispanic White students (*M* = 76.97, *SD* = 15.19) had significantly higher levels of physical quality of life than students of other racial or ethnic backgrounds (*M* = 73.71, *SD* = 17.13), *t*(328.30) = 2.45, *p* = .015, *d* = 0.27. Fourth year medical students had significantly higher levels of physical quality of life when compared to first year (*p* < .001), second year (*p* = .002), and third year students (*p* < .001).

## Discussion

The purpose of this investigation was to provide an initial examination of the effects of self-care on perceived stress and quality of life in U.S. medical students. Our data showed a strong inverse relationship between perceived stress and medical students’ quality of life, and suggest that student self-care may help buffer this relationship (Fig. 1). These initial findings suggest that students who report increased engagement in self-care may be utilizing a positive strategy for reducing the effects of stress on quality of life in U.S. medical students; those who reported using a variety of health promotion practices within several domains simultaneously also reported less stress and higher quality of life. A multifaceted approach to self-care that includes nutrition, interpersonal relations, health responsibility, physical activity, spiritual growth, and stress management [10] may thus collectively deter the consequences of stress on quality of life by reducing the strength of the relationship between these two variables.

The perceived stress levels reported by the current sample supported previous findings that 50% of medical students scored at least half of a standard deviation greater than the norm for age-matched peers in the U.S. general population [38]. In the current study, 56.5% of respondents endorsed scores of 25 or higher (*M* = 21.1, *SD* = 7.2 for age-matched peers [33]), meaning over half of the students in the sample reported stress levels substantively higher than that of age matched peers in the U.S. population. Although high in comparison to the general population, such elevations on the Perceived Stress Scale are not uncommon and have been found in other samples, including doctoral level pharmacy students [39, 40], Physician Assistant students [40]; doctoral level psychology students [41], and practicing health service professionals (i.e., dietician/nutrition professionals, nurses, physicians, social workers) [42].

Consistent with the literature on medical student distress, our study recognized significant demographic differences in student self-care, stress, and quality of life [43–45]. Non-Hispanic White students reported significantly lower levels of stress, higher engagement in self-care, and higher physical quality of life in comparison to peers from other racial and ethnic backgrounds. These findings have practical implications for medical education and reinforce the need for further research. Findings in future studies can help identify the underlying causes of these observed differences and inform effective interventions to improve the well-being of the increasingly diverse U.S. medical student population [2].

Although senior students reported better self-care and less stress, third year-students’ self-care practices were more impoverished than second and fourth year students. This finding is consistent with previous studies showing a decline in well-being during the third year and reinforces the necessity of education on self-care, and time to implement it, well before students’ first clinical experiences [43]. Although men were more likely to report poorer self-care, women in our study experienced significantly more stress than men. As previous research indicates increased levels of distress for women over the course of medical training [46–51], our findings further implore institutions to address the unique stressors of women in medicine as well as the need to understand what inhibits men from more actively engaging in self-care.

An abundance of literature exists on individual ways to improve medical student stress [8, 9, 20–26]. Although our study demonstrates the validity of a broader, multi-component operationalization of effective self-care, further research is needed to understand the extent to which the different dimensions of self-care interact and combine with one another to impact medical student quality of life. Participants who reported higher engagement in self-care activities may also see themselves as readily taking steps to regain resilience and improve their ability to fully participate in their education during times of heightened distress, reflecting a set of positive personality traits that is highly desirable in medical education (e.g., low harm avoidance, high persistence, self-directedness, cooperativeness [52]). Future investigations may seek to identify specific factors that account for higher and lower levels of reported engagement in self-care behaviors for US medical students.

Recognizing that resilience of students may also be tied to the perception of one’s learning environment [53, 54], future researchers may choose to examine the extent to which institutions can encourage students to increase engagement within different combinations of the six factors of self-care. Continued research regarding the exploration and implementation of institutional strategies is warranted. It is also important that medical institutions continue to explore new and less programmed ways to reduce medical student stress and improve quality of life, as suggested by recent mixed methods research investigating the verbatim opinions of students on their own unique self-care practices [11].

As expected, participants in our study showed little variation in their tendency to indicate poorer quality of life when their perceived stress scale responses were higher (Table 1). Although our findings are consistent with other studies that have shown a strong inverse relationship between measures of perceived stress and both physical and mental quality of life in medical students [38], our findings at the same time suggest the added possibility that self-disclosure of more robust self-care during medical school may diminish the strength of this relationship (Fig. 1). However, student stress cannot be avoided during medical education and in fact may not in all circumstances be deleterious [55, 56]. Hence, a causal relationship between high levels of perceived stress and poor quality of life during medical education cannot be determined from cross-sectional studies utilizing self-report measures at a single point in time. For example, sudden spikes in stress often herald important developmental transitions and thus high levels of stress during medical education may not always coincide with poorer quality of life [57–59] or be affected by changes in self-care behaviors. One interpretation of our findings suggests that for medical students, the higher stress levels typically associated with both abnormal levels of psychological distress and normal developmental processes, if paired with positive engagements in self-care, may be more likely to facilitate healthier negotiations of these challenging periods.

## Limitations

Perhaps the most salient limitation to this investigation pertains to the sample size. Although we recruited enough participants to secure adequate statistical power for the study, the sample represents approximately 1% of medical students in the U.S. [60]. Moreover, only one third of medical schools in the U.S. were included in the study, and some of the schools in the sample were represented by only a few students. Therefore, there was not an even distribution across medical schools that participated. It is also unclear whether the 49 institutions represented in the sample provide programming or wellness programs for students, or what percentage of students engage in such programs. Given these limitations, one must be careful not to generalize findings to all medical students in the US or outside of the US, and rather, to inform future research with more representative samples.

All measures were self-report and based on initial self-perceptions, making our results susceptible to social desirability and recall bias. The personal circumstances and stress levels of our participants may also have affected their willingness to participate and thus our data may reflect students with lower or higher levels of stressful conditions relative to the total sample of U.S. medical students. Additionally, women and Non-Hispanic White medical students were over represented in our sample when compared to data from 2015 from the Association of Medical Colleges (AAMC) (60.1% vs. 47% and 71.2% vs. 53%, respectively) [60]. Finally, the use of the respondent driven sampling method [30] prevents us from being able to assess how many students received the call for participation, thus preventing us from being able to calculate an accurate response rate.

Responses from participants in this study reflected only individual factors affecting self-care in medical students. Building on previous work in this area, future investigations that include the important role of institutional factors in medical student distress and well-being may provide a more complete understanding of the complex relationships between stress, quality of life, and staying well during medical education and the role of self-care in facilitating a positive outcome for emerging physicians [61–64]. Despite these limitations, our results provide a foundation on which to further consider the impact of self-care on students’ perceptions of stress and self-reported quality of life during medical education.

## Conclusions

Using a multi-dimensional model of self-care, our study identified significant interactions between medical student stress, reported engagement in self-care and both physical and psychological quality of life. These findings suggest that US medical students who report high engagement in a wide array of self-care activities may experience a protective effect on the negative relationship between stress and quality of life. Yet despite the promise of more effective strategies for stress reduction in medical students, current research indicates that students continue to encounter stigma, time constraints, and other barriers that frequently prevent them from seeking the help they need [14, 43]. Further investigations using a larger national or international cohort are necessary to better understand the complex relationships between these variables at various stages of medical education. Medical educators are in a prime position to redirect this pattern. Knowing that the strength and breadth of medical students’ own health promotion practices may directly affect the relationship between stress and quality of life during medical education provides further evidence that our work toward improving medical student self-care is an important area of medical education.

## Abbreviations

AAMC: Association of American Medical Colleges
HPLPII: Health Promoting Lifestyle Profile II
IP: Internet Protocol
IRB: Institutional Review Board
LCME: Liaison Committee on Medical Education
NIH: National Institutes of Health
WHO: World Health Organization
